# Supplementary material for: Transgenic Sweet Orange Expressing the Sarcotoxin IA Gene Produces High-Quality Fruit and Shows Tolerance to ‘Candidatus Liberibacter asiaticus’
Source: Int J Mol Sci. 2022 Aug 18;23(16):9300. doi: 10.3390/ijms23169300 (PMC9409437; doi:10.3390/ijms23169300)
Supplement: Supplementary file 1 [file ijms-23-09300-s001.zip › ijms-1811850-supplementary.pdf]

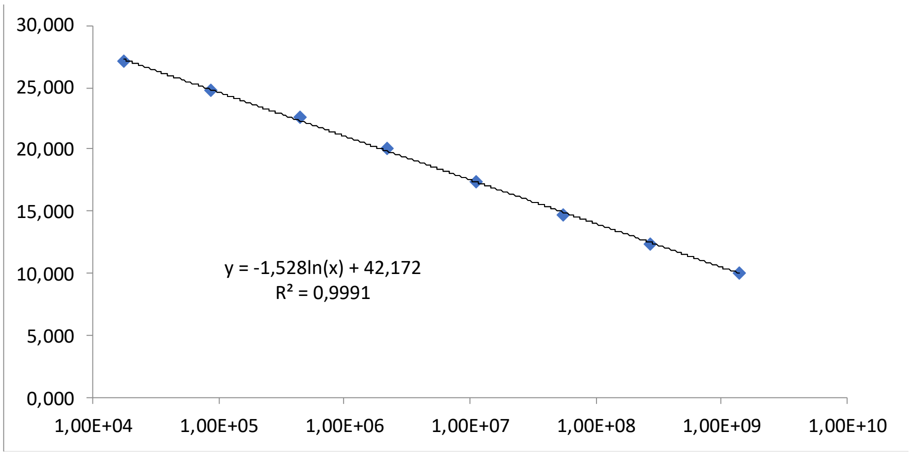

Figure S1. Calibration standard curve obtained from eight known concentrations of the pST10 plasmid containing the *stx IA* gene. Ct values in a logarithmic scale correspond to the initial copy number of the sequences encoding the *stx IA* gene (number of copies per μL).

Table S1. RT-qPCR quantification of *stx IA* gene expression (Ct-value) in the Pera sweet orange transgenic lines STX-5, STX-11, STX-12, and STX-13, based on cDNA synthesized from total RNA. Total RNA of a non-transgenic plant was also included as a negative control.

STX IA transgenic lines	Threshold cycle (Ct-value)
Control	00.00
STX-5	21.29
STX-11	19.88
STX-12	19.18
STX-13	21.26

Table S2. RT-qPCR quantification of '*Candidatus* Liberibacter asiaticus' (*C*Las; Ct-value) in the Pera sweet orange transgenic lines (STX-5, STX-11, STX-12, and STX-13) and non-transgenic (control), based on cDNA synthesized from total RNA.

STX IA transgenic lines	Threshold cycle (Ct-value)	Number of *C*Las copies per gram (Log_{10})
Control	28.78	6.630
STX-5	21.42	8.748
STX-11	22.64	8.396
STX-12	21.48	8.730
STX-13	25.03	7.994

Table S3. List of the primer sequences used to identify the STX IA transgene (STX IA F/STX IA R) and the '*Candidatus* Liberibacter asiaticus' (CLas) (A2/J5; Oi1/Oi2c) in the Pera sweet orange (*Citrus* × *sinensis*) plants.

**Primer set**	**Primer sequence (5′–3′)**	**Amplicon size (bp)**
STX IA F/STX IA R	GCC TCG GAT CCA TTC AAG ATA CAA CAT TTC T/AAC TCG AGC TCA TTT ATC TGG CTG TAG CAG CAA CAT	268
A2/J5	TAT AAA GGT TGA CCT TTC GAG TTT/ ACA AAA GCA GAA ATA GCA CGA ACA A	703
Oi1/Oi2c	GCG CGT ATG CAA TAC GAG CGG CA/ GCC TCG CGA CTT CGC AAC CCA T	1,160
